# A Notable Anniversary

**Published:** 1919-07-19

**Authors:** 


### SIR WILLIAM OSLER'S SEVENTIETH BIRTHDAY.

### A NOTABLE ANNIVERSARY.

It was our misfortune to be prevented from attending the presentation by Sir Clifford Allbutt to Sir William Osler in the Barnes Hall of the Royal Society of Medicine on the 11th instant. It was nearly forty years ago, in Canada, that we first had the good fortune to come across Sir William Osler and got our first insight into the man and his measure. It has been our habit to visit the United States and Canada often in the decades of years which have intervened, and in this way, and in the course of our work, we have had the privilege of learning of, seeing, and appraising many things and much of the work which our treasured friend has taken part in, or made entirely his own. It is a splendid record and an uplifting example of what one man may accomplish with God's blessing, if he devotes himself whole-heartedly and continuously to the next thing, whatever it may be, which is brought within the confines of his life-work and efforts, throughout many years.

Of his student days we know nothing personally, save that Sir William himself and his life are a guarantee that he was a strenuous student, made fertile by wide experience and diversity, for this section of his career included Toronto, Montreal, London, Berlin, and Vienna. When we remember him at home in the Western Hemisphere, as Professor of the Institute of Medicine, McGill University, Montreal, 1874-84, a return to that University to-day demonstrates that the effects of his teaching, its example, and the impetus which they gave to McGill have endured, and will endure throughout its history, to the great advantage of every student who may be trained there for his life's work. Next comes Osler's introduction to Philadelphia, as Professor of Clinical Medicine at the University of Pennsylvania, five strenuous years, where he had the companionship of notable men of science, amongst them Weir Mitchell and John Billings, of blessed memory to all who had the privilege of knowing them. Next, Baltimore is given a University and a remarkable, modern, up-to-date Hospital, the Johns Hopkins, which latter was John Billings' great production. At the time it was built this hospital and the University attached to it were full of interest and points of importance to all hospital administrators. Could anything be more fortunate or admirable than that Professor Osler, as he then was, should become Professor of Medicine to the Johns Hopkins University from 1889 onwards, until he came to Oxford as Regius Professor of Medicine in 1905? No one who has had the privilege of visiting Baltimore and spending time in its University and Hospital, much more if they did it, as we have done, on several occasions, can ever forget the joy and inspiration received, or the burning desire exhibited by all workers there, each to spread the knowledge and extend the system of this magnificent centre of medical treatment and scientific development throughout the world. Many changes have been made and improvements and extensions introduced in later years, but the work accomplished at Baltimore has been so sound, excellent, and uplifting, and the influence of our friend the Professor of Medicine so health-giving, energising, and important, that it spread within the University itself in all directions. Every one of the men who had the privilege of entering into the atmosphere and spirit which centred round Osler and his *confrères* and colleagues at this notable seat of learning has cause for gladness. Certainly the Johns Hopkins University and Hospital under the continuous guidance and help of able men, its professors, teachers, and administrators, should make its mark in the development of the new world, which civilisation looks forward to, through the League of Nations, and the unity of civilised mankind.

